# Diagnostic method of mass spectrometry for detecting lymph node metastasis of non‐small cell lung cancer

**DOI:** 10.1111/1759-7714.15179

**Published:** 2023-12-11

**Authors:** Ryuichi Yoshimura, Wataru Shigeeda, Yuji Fujita, Tetsuo Kokaji, Hiroyuki Deguchi, Makoto Tomoyasu, Satoshi Kudo, Yuka Kaneko, Hironaga Kanno, Hidenobu Iwai, Tomohiko Mase, Hajime Saito

**Affiliations:** ^1^ Department of Thoracic Surgery Iwate Medical University Iwate Japan; ^2^ Department of Critical Care and Disaster Medicine Iwate Medical University Iwate Japan; ^3^ SCIEX, 4‐7‐35 Kitashinagawa Tokyo Japan

**Keywords:** lung cancer, lymph node, mass spectrometry

## Abstract

**Background:**

Histopathology by pathologists is essential in the diagnosis of non‐small cell lung cancer (NSCLC). However, auxiliary diagnostic procedures for malignant tumor have continued to evolve. Despite the poor prognosis of patients with NSCLC, the application of the latest procedures and technologies to the field of lung cancer has lagged. Mass spectrometry was used to detect trace amounts of peptides in human tissue with high accuracy. The aim of this study was to establish a method for diagnostic mass spectrometry to identify lymph node metastasis by detecting cytokeratin (CK)19, a useful biomarker in lung cancer.

**Methods:**

We collected 81 lymph nodes with positive expression of CK19 in patients who underwent radical surgical resection in the Department of Thoracic Surgery at Iwate Medical University between May 2020 and December 2022. An X500R instrument was used for sample analysis. A positive result for lymph node metastasis as the detection at least two product ions (FGPGVAFR and ILGATIENSR) from CK19 was defined.

**Results:**

Our study indicated a high diagnostic efficiency for mass spectrometry, with 87.5% sensitivity and 91.2% specificity. The mutual concordance of mass spectrometry methods and histopathological diagnosis was 90.1%.

**Conclusions:**

Mass spectrometry offers high diagnostic accuracy and can be clinically applied to auxiliary diagnostic procedures for lymph node metastasis from NSCLC.

## Graphical abstract

Mass spectrometry holds potential as an auxiliary diagnostic procedure for detecting lymph node metastasis in patients with NSCLC.

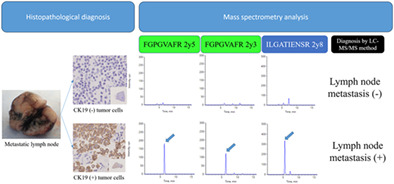

## INTRODUCTION

Lung cancer is a common malignancy that has high mortality and has become a public health concern all over the world. An estimated 1.8 million deaths were attributed to lung cancer in 2020.^1^, ^2^ Non‐small cell lung cancer (NSCLC) is the main histological subtype of lung cancer, accounting for approximately 80% of cases in Japan.^3^ Anatomical lobectomy with mediastinal lymph node dissection has been the gold standard of surgical treatment for early‐stage NSCLC.^4^ Even after curative resection, 30%–55% of NSCLC patients develop recurrence and die of the disease.^5^ The unfavorable prognosis and recurrence of NSCLC are considered to be closely associated with lymph node metastasis. Moreover, lymph node metastasis plays a key role in the staging process, which is directly linked to therapeutic strategies against NSCLC. Segmental pulmonary resection has been reported as superior to pulmonary lobectomy in aggressive reduction surgery for peripheral early lung cancers of 2 cm or less, and the importance of intraoperative diagnosis for lymph node metastasis is increasing (JCOG0802/WJOG4607L).^6^ Accurate evaluation and diagnosis of resected lymph nodes thus appears extremely important for the treatment of NSCLC.

To date, histopathological methods using hematoxylin and eosin (HE) staining have been the primary approach used in practical procedures for detecting tumor cells in hilar and mediastinal lymph nodes. The sensitivity of this morphological examination can be increased using immunohistochemistry (IHC). Regardless of progress in histopathological methods, the major problem of detecting micrometastases, small tumor cell populations or solitary tumor cells in lymph node persists. Corner et al. described the misidentification rates of up to 20% in tumor cells located outside lymph nodes using histopathological methods.^7^ Furthermore, histopathological procedures are occasionally challenging for frozen sections or small crushed samples.

In recent years, some researchers have reported the utility of mass spectrometry (MS) for diagnosing malignant tumors such as renal cell carcinoma and hepatocellular carcinoma.^8^, ^9^ MS was established in the field of nature science approximately half a century ago, with detection of identical chromatograms for a wide range of substances from tissue samples.^10^ Liquid chromatography–tandem mass spectrometry (LC–MS/MS) have been developed as an innovative tool for the analysis of small samples, offering improved specificity over traditional methods. MS imaging and probe electrospray ionization (PESI)‐mass spectrometry have been described as useful tools for diagnosing lung cancer or distinguishing between normal and cancerous tissues.^11^, ^12^ PESI‐MS allows rapid evaluation and is extremely useful, but has the weakness of being able to measure only that part of the specimen obtained by puncture biopsy. However, diagnostic procedures to detect specific substances by LC–MS/MS in the field of lung cancer have rarely been reported. Novel molecular techniques to identify targeted substances in human biological tissues could potentially improve the accuracy of diagnosis for lung cancer. The purpose of this study was to establish a molecular diagnostic LC–MS/MS method that could discriminate lymph node metastasis by detecting CK19 as a useful biomarker for lung cancer. To the best of our knowledge, this study represents the first report on identification of CK19 in human hilar or mediastinal lymph nodes using LC–MS/MS.

## METHODS

### Patient selection

We reviewed the medical records of patients who underwent radical pulmonary resection for NSCLC with clinical node‐positive results in the Department of Thoracic Surgery at Iwate Medical University between May 2020 and December 2022. This study was approved by the institutional review board (approval no. MH2020‐233) and informed consent was obtained from all patients. No patients received preoperative chemotherapy or radiation. A total of 81 lymph nodes from 37 patients were enrolled for analysis in this study.

### Preoperative examination and management

All patients were given a complete preoperative pulmonary evaluation. Clinical staging was based on chest x‐ray, contrast‐enhanced computed tomography (CT) of the chest and abdomen, brain CT or magnetic resonance imaging (MRI) and positron emission tomography (PET). All cases underwent the above examinations and evaluation of clinical staging. The consolidation component was defined as an area of increased opacity that completely obscured underlying vascular markings. The ground‐glass component was defined as an area of slight, homogeneous increase in density that did not the obscure underlying vascular markings.^9^ The consolidation‐to‐tumor ratio was defined as the ratio of the maximum diameter of the consolidation component to maximum tumor diameter in this study. Pathological staging was decided based on the eighth edition of the American Joint Commission on Cancer TNM staging system.^13^ Histological classification was based on the World Health Organization criteria.^14^

### Surgical procedures

Complete video‐assisted thoracic surgery (VATS) was performed via three ports under monitor vision only. Pulmonary segmentectomy, lobectomy, bilobectomy or pneumonectomy and systematic dissection of hilar and mediastinal lymph nodes were performed in all cases.

### Sample preparation

Resected lymph nodes were immediately deep frozen (−80°C) until analysis. Lymph nodes were split in half, with one half used for HE and IHC examinations for CK19, and the other half for analysis by LC–MS. Frozen tissue samples were sliced into 5‐ to 10 mg sections and homogenized in RIPA lysis buffer (Nacalai Tesque) on ice. The homogenate was centrifuged at 10 000 × *g* for 10 min at 4°C. A 10‐μL aliquot of supernatant was added to 40 μL of 6 M Guanidine (Nacalai Tesque), 1 μL of reducing reagent, 500 mM of dithiothreitol (Fujifilm Wako Pure Chemical) and 1 μL of 1 M Tris–HCl (Cosmo Bio), then incubated at 60°C for 30 min. This solution with the addition of 2 μL of 500 mM iodoacetamide (Fujifilm Wako Pure Chemical) was incubated at room temperature for 30 min in the dark for alkylation. The alkylated solution was added to 100 μL of sodium dodecyl sulfate (SDS) remover (Invent Biotechnologies) for removal of SDS in the solution, and the solution including SDS remover was centrifuged at 14000 × *g* for 5 min at 4°C. The supernatant was adjusted to pH 8 with the addition of 200 mM Tris–HCl. The pH‐adjusted solution was added to 5 μL of 1.0 μg/μL trypsin solution (Promega). Trypsin digestion was performed at 37°C overnight, then stopped by the addition of 10 μL of 10% trifluoroacetic acid, and a total fluid volume of 500 μL was achieved by the addition of H_2_O. Finally, the solution was purified by solid‐phase extraction using a MonoSpin C18 column (GL Sciences). The column was conditioned with 200 μL of 0.2% trifluoroacetic acid aqueous solution at 2000 × *g* for 1 min. The sample solution (200 μL) was applied to the conditioned column, and the column was centrifuged for 1 min at 2000 × *g*. The column was then washed using 200 μL of 0.2% trifluoroacetic acid aqueous solution. Analytes were then eluted with 200 μL of ethanol/H_2_O/trifluoroacetic acid (60:40:0.1) for 1 min at 2000 × *g*. The eluted solutions were injected to the LC–MS/MS.

### LC–MS/MS system

An ExionLC AC system (AB SCIEX) was used as the LC system. Chromatographic separation was achieved under gradient condition using a Waters Acquity UPLC CSH C18 column (2.1 mm 150 mm, 1.7 μm). The mobile phases were water with 0.1% formic acid (A) and acetonitrile with 0.1% formic acid (B). Samples were run for 12.5 min at a flow rate of 0.25 mL/min with the following gradient program for solvent B: 0–0.5 min, 3% hold; 0.5–8 min, a linear increase to 65%; 8–10 min, a linear increase to 90%; 10–12.5 min, 90% hold. Column oven temperature was 40°C and the injection volume was 30 μL.

The LC system was interfaced with an X500R QTOF system (AB SCIEX) equipped with a Turbo‐V IonSpray. The mass spectrometer was operated in electrospray ionization mode. MS source conditions were optimized as follows: curtain gas, 30 psi; collision gas, 7; nebulizer gas, 30 psi; heater gas, 40 psi; ion spray voltage, 5500 V in positive ion mode; and source temperature, 300°C. Determination of CK19 was achieved using high‐resolution multiple reaction monitoring with the LC–MS/MS. Transitions were as follows: FGPGVAFR, m/z 425.73 > m/z 549.31 and m/z 425.73 > m/z 393.22; ILGATIENSR m/z 537.3 > m/z 847.43.

### Statistical analysis

JMP version 14.1.0 statistical software (SAS Institute) was used for all statistical analyses in this study. Categorical variables were compared between groups using Pearson's chi‐square test or the Wilcoxon rank‐sum test. To estimate the sensitivity and specificity of diagnostic procedures, the LC–MS method was compared to the traditional histopathological method. Differences between groups were considered significant for values of *p* < 0.05. Continuous data as mean, and categorical data are expressed as count and percentage.

## RESULTS

A total of 84 lymph nodes obtained from 37 patients who underwent radical pulmonary resection for NSCLC were analyzed. Of these, three lymph nodes that were negative for CK19 in IHC were excluded. Finally, a total of 81 lymph nodes were analyzed with LC–MS/MS. Clinical characteristics of the study population are summarized in Table 1. The cohort comprised 37 patients (30 men, 7 women) with a median age of 68.2 years (range, 42–82 years). Radiological examination showed a median tumor size of 33.3 mm, consolidation‐to‐tumor ratio was 99.6% and median maximum standardized uptake value on ^18^F‐fluorodeoxyglucose PET was 9.4. Regarding surgical procedures, lobectomy was performed for 89.2% of patients. In terms of the pathological subtype of NSCLC, adenocarcinoma was observed in 76.5% of primary tumors and squamous cell carcinoma in 17.7%. For resected lymph nodes, pN1 was observed in 37.8%, pN2 was in 27.0%.

**TABLE 1:** Patient characteristics.

Variables	
Number of patients	37
Number of lymph nodes	81
Age (years)	68.2 ± 7.7
Sex	
Male	30 (81.1%)
Female	7 (18.9%)
Brinkman index	663.2 ± 474.6
Radiological findings	
Tumor size (mm)	33.3 ± 12.0
Consolidation/tumor ratio (%)	99.6
Maximum standardized uptake value	9.4 ± 6.3
Tumor side	
Right	18 (48.6%)
Left	19 (51.4%)
Tumor location	
Upper lobe	22 (59.5%)
Middle lobe	2 (5.4%)
Lower lobe	13 (35.1%)
Clinical T stage	
T1a	7 (18.9%)
T1b	5 (13.5%)
T2a	19 (51.4%)
T2b	4 (10.8%)
T3	2 (5.4%)
Surgical procedure	
Segmentectomy	2 (5.4%)
Lobectomy	33 (89.2%)
Bilobectomy	1 (2.7%)
Pneumonectomy	1 (2.7%)
Pathological subtype	
Adenocarcinoma	25 (76.5%)
Squamous carcinoma	11 (17.7%)
Cartinoid	1 (5.9%)
Number of dissected lymph nodes	20.5 ± 9.8
Lymph node metastasis	
pN0	13 (35.1%)
pN1	14 (37.8%)
pN2	10 (27.0%)

**FIGURE 1:**
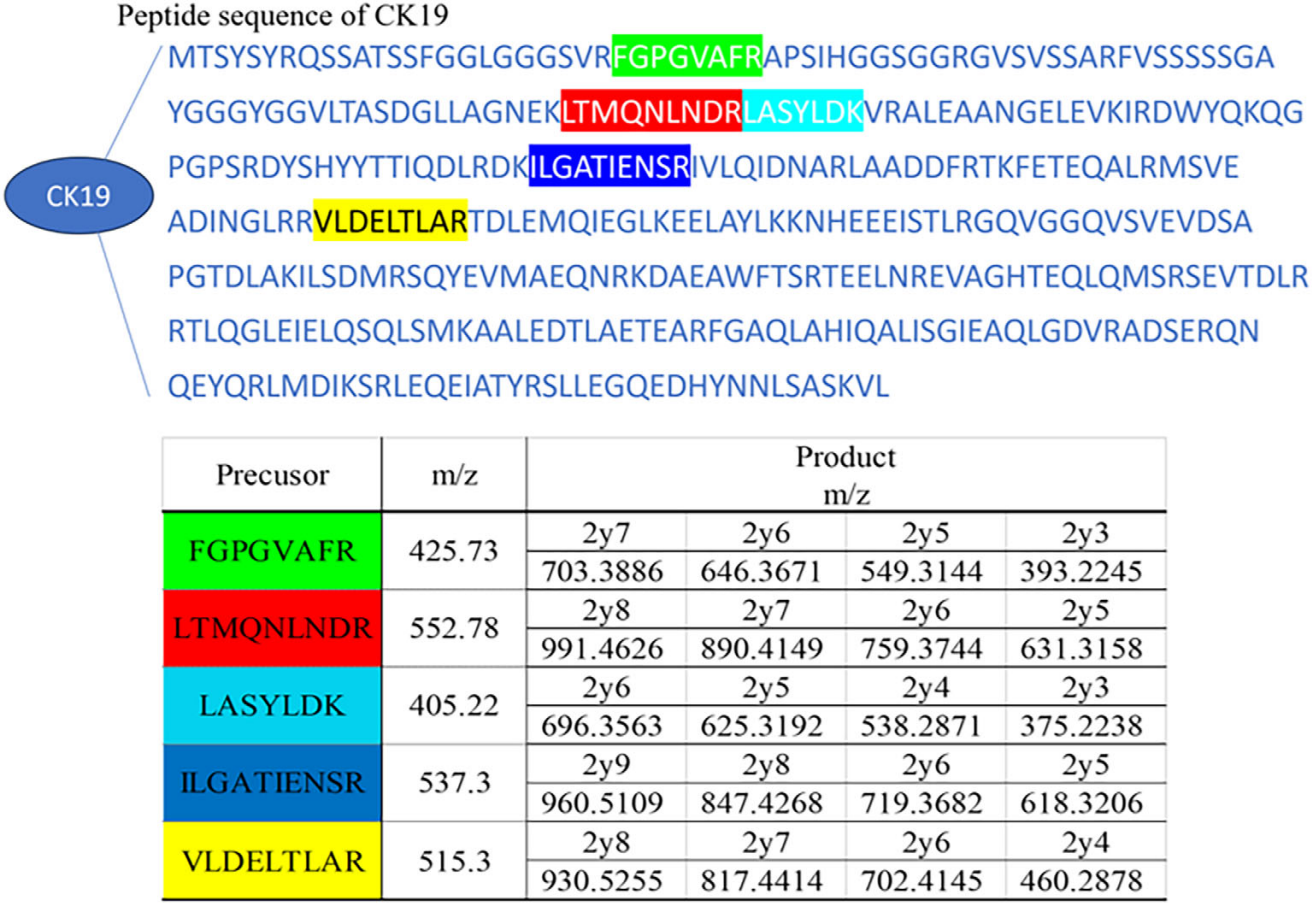
Candidate peptides for CK19 sequence. These peptides were obtained using Protein Pilot. Transitions were determined using Skyline (freely available online at http://skyline.maccosslab.org).

**FIGURE 2:**
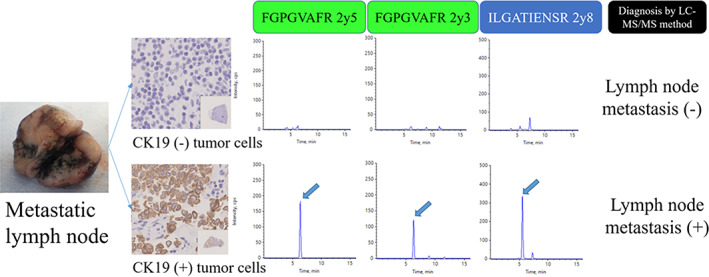
Definitions of CK19 expression in this study.

Figure 1 shows candidate‐specific peptides obtained from the sequences of CK19. Of the five peptides, two (FGPGVAFR and ILGATIENSR) were detected in lymph node metastasis using the developed LC–MS/MS method. On the other hand, samples from lymph nodes without metastasis or CK19‐negative tumors did not show detection of these peptides by LC–MS/MS analysis (Figure 2). In this study, a positive result for lymph node metastasis was defined as detection of FGPGVAFR and ILGATIENSR in the lymph node sample. Table 2 shows the comparison of tumor metastasis diagnoses using the LC–MS/MS and histological methods. Diagnosis of tumor metastasis using LC–MS/MS offered 87.5% sensitivity and 91.2% specificity. The concordance rate of these diagnostic methods was 90.1%. Table 3 shows profiles of the eight samples with discordance between results from LC–MS/MS and histological diagnoses. These samples all represented adenocarcinoma according to pathological diagnosis. Of these eight samples, three showed positive results from histological diagnosis and negative results from LC–MS/MS diagnosis. The remaining five samples showed negative results from histological diagnosis (pathological N0) and positive results from LC–MS/MS diagnosis. Of these five samples, two samples presented multiple station N2.

**TABLE 2:** The two by two classification table in this study.

	Pathological diagnosis		Total
	Metastasis (+)	Metastasis (−)	
LC‐MS diagnosis			
Metastasis (+)	21	5	26
Metastasis (−)	3	52	55
Total	24	57	81

**TABLE 3:** The consequences of discordances.

Patient No	Lymph node	Pathological subtype	Histological diagnosis	Mass spectrometry diagnosis	Number of metastatic lymph nodes	Multiple Station N2
1	12u	Adenocarcinoma	Positive	Negative	1	
2	4R	Adenocarcinoma	Positive	Negative	5	
3	12u	Adenocarcinoma	Positive	Negative	0	
4	2R	Adenocarcinoma	Negative	Positive	1	
5	11	Adenocarcinoma	Negative	Positive	10	〇
6	7	Adenocarcinoma	Negative	Positive	1	
7	11s	Adenocarcinoma	Negative	Positive	3	
8	11s	Adenocarcinoma	Negative	Positive	13	〇

## DISCUSSION

The purpose of this study was to establish a molecular diagnostic LC–MS/MS method to identify lymph node metastasis of NSCLC by detecting CK19. The present outcomes demonstrated that the LC–MS/MS method described allows highly precise prediction of pathological lymph node metastasis. Cytokeratins belong to the family of intermediate filament proteins in the human body.^15^ Among these, CK19 has been identified as most abundant in human epithelial cells. As 88.0% of NSCLCs reportedly express CK19, this protein is widely used as a tumor marker for NSCLC and is widely applied for evaluating response to therapy.^16^ In the present study, the rate of CK19 expression was 96.4% in the 84 analyzed samples. For these reasons, we selected CK19 as a marker of lymph node metastasis.

Molecular detection of CK19 has been suggested as a method for detecting NSCLC metastasis. In particular, one‐step nucleic acid amplification (OSNA) is considered a representative method.^17^, ^18^, ^19^, ^20^ OSNA is a molecular detection technique that can quantify levels of CK19 mRNA to reflect the presence of tumor cells. This method uses reverse transcription loop‐mediated isothermal amplification to calculate CK19 mRNA expression in tumor cells. Previous studies using this method have compared the OSNA method with traditional histopathological methods using HE in NSCLC patients. In those studies, OSNA was reported to offer 79.7%–100% sensitivity and 91.7%–98.5% specificity.^17^, ^18^, ^19^, ^20^ In the present study, although only two peptides were used to detect CK19, the LC–MS/MS method developed offered 87.5% sensitivity and 91.2% specificity, comparing favorably with OSNA. These outcomes suggest that mass spectrometry provides sufficient diagnostic accuracy and can allow auxiliary diagnosis of lymph node metastasis of NSCLC. The advantages of this method are: (1) measurement is possible even with minute amounts of sample; (2) opportunities for examination rather than diagnosis because as an objective and no requirement of special skills; (3) differentiate multiple patterns in one analysis; and (4) the time for analysis can be shortened by narrowing the analysis windows, potentially allowing application to rapid intraoperative diagnosis.

With reference to unexpected findings, five lymph nodes from five patients showed discordant results, LC–MS/MS yielded a positive result where histopathological examination diagnosed a negative finding. Table 3 shows the clinical and pathological features of these false‐positive cases. Several possibilities could explain such results. First, the slice used for LC–MS/MS analysis might not have contained tumor cells. When preparing sample sections, we sliced the lymph node into pieces of approximately 10 mg. Slice sections could show allocation bias as described by other researchers.^18^, ^20^ Second, the LC–MS/MS method has potential to detect CK19 with high sensitivity, allowing identification of phenomena such as micrometastasis. In fact, two of five false‐positive cases were multiple N2 patients. On the other hand, three lymph nodes were diagnosed as negative by LC–MS/MS but positive on histopathological examination. The main reason for false‐negative results may be ion suppression effects induced by the matrix from the biological sample. Further cleanup in sample preparation might thus be needed.

The main limitation of the present LC–MS/MS method in this study was the focus on only CK19‐positive tumors. While CK19 is highly expressed in NSCLC, approximately 10% of NSCLC are known to be negative for CK19. Masai et al. reported that some lung cancer subtypes showed low rates of CK19 positivity, such as 54.8% in pleomorphic carcinoma, 54.5% in large cell carcinoma, and 34.0% in carcinoid tumor.^16^ Further study and careful diagnosis are thus required for the diagnosis of several tumor subtypes. This same limitation has been mentioned in reference to OSNA.^18^, ^21^ However, LC–MS/MS can detect multiple peptides in a single analysis, offering potential for the diagnosis of CK19‐negative NSCLC if suitable additional markers can be identified. Further investigation of other biomarkers for NSCLC is needed to address the issue of CK19‐negative tumors. Additionally, this study is a retrospective nature and small number of patients from a single institute. Therefore, all findings may not always be extrapolated to other institutions.

In conclusion, mass spectrometry appears to hold potential as an auxiliary diagnostic procedure for detecting lymph node metastasis in patients with NSCLC.

## AUTHOR CONTRIBUTIONS

All authors had full access to the data in the study and take responsibility for the integrity of the data and the accuracy of the data analysis. Study concept and design: Ryuichi Yoshimura and Hajime Saito. Acquisition of data: Ryuichi Yoshimura, Wataru Shigeeda, Yuji Fujita, Tetsuo Kokaji, Hiroyuki Deguchi, Makoto Tomoyasu, Satoshi Kudo, YukaKaneko, Hironaga Kanno and Hidenobu Iwai. Analysis and interpretation of the data: Ryuichi Yoshimura, Wataru Shigeeda and Yuji Fujita. Drafting of the manuscript: Ryuichi Yoshimura and Hajime Saito. Criticalrevision of the manuscript for important intellectual content: Ryuichi Yoshimura and Hajime Saito. Statistical analysis: Ryuichi Yoshimura and Hajime Saito. Obtained funding: Hiroyuki Deguchi and Hajime Saito. Administrative, technical and material support: Yuji Fujita and Tetsuo Kokaji.

## CONFLICT OF INTEREST STATEMENT

The authors report no conflicts of interest.
